# A Quantum-Hybrid Framework for Urban Environmental Forecasting Integrating Advanced AI and Geospatial Simulation

**DOI:** 10.3390/s25247422

**Published:** 2025-12-05

**Authors:** Janis Peksa, Andrii Perekrest, Kyrylo Vadurin, Dmytro Mamchur

**Affiliations:** 1 Information Technology Faculty, Turiba University, Graudu Street 68, LV-1058 Riga, Latvia; janis.peksa@turiba.lv (J.P.); pksg13@gmail.com (A.P.); 2 Institute of Information Technology, Riga Technical University, Kalku Street 1, LV-1658 Riga, Latvia; 3 Computer Engineering and Electronics Department, Kremenchuk Mykhailo Ostrohradskyi National University, Universitetska Street 20, 39600 Kremenchuk, Ukraine; kir3337@gmail.com

**Keywords:** forecasting, environmental monitoring, quantum computing, deep neural networks, time series, spatial modeling, LSTM, AutoARIMA, DPPDM, environmental information system

## Abstract

The paper examines the development of forecasting and modeling technologies for environmental processes using classical and quantum data analysis methods. The main focus is on the integration of deep neural networks and classical algorithms, such as AutoARIMA and BATS, with quantum approaches to improve the accuracy of forecasting environmental parameters. The research is aimed at solving key problems in environmental monitoring, particularly insufficient forecast accuracy and the complexity of processing small data with high discretization. We developed the concept of an adaptive system for predicting environmental conditions in urban agglomerations. Hybrid forecasting methods were proposed, which include the integration of quantum layers in LSTM, Transformer, ARIMA, and other models. Approaches to spatial interpolation of environmental data and the creation of an interactive air pollution simulator based on the A* algorithm and the Gaussian kernel were considered. Experimental results confirmed the effectiveness of the proposed methods. The practical significance lies in the possibility of using the developed models for operational monitoring and forecasting of environmental threats. The results of the work can be applied in environmental information systems to increase the accuracy of forecasts and adaptability to changing environmental conditions.

## 1. Introduction

The escalating pace of global urbanization concentrates populations in dense metropolitan areas, intensifying the need for accurate, high-resolution environmental monitoring. Urban atmospheres are complex systems governed by the intricate interplay of natural meteorological phenomena and a multitude of anthropogenic activities, resulting in a high-dimensional, non-linear dynamic environment [1]. The timely prediction of air quality parameters—such as pollutant concentrations, temperature, and humidity—is paramount for mitigating public health risks, informing sustainable urban planning, and executing effective environmental policies [2]. Achieving this requires a paradigm shift from static monitoring to dynamic, predictive environmental intelligence.

A promising paradigm for this shift is the Urban Digital Twin, a concept that involves creating a dynamic, virtual replica of a city by integrating real-time sensor data, predictive models, and interactive simulations [3]. Such a system can provide unprecedented insights into urban environmental processes, enabling proactive management and “what-if” scenario analysis. The development of a robust Urban Digital Twin hinges on the ability to process vast, heterogeneous data streams and to model their complex, often non-linear, interdependencies. Existing Urban Digital Twin frameworks often focus heavily on static visual representation or architectural data, lacking dynamic, diverse predictive engines capable of processing real-time sensor data [4]. While studies such as Verde et al. [4] demonstrate the utility of digital simulation for evaluating climate adaptation strategies, the need for frameworks that tightly couple these simulations with advanced AI-driven forecasting agents still exists.

Presented research addresses the critical challenge of converting raw, high-dimensional data streams from urban sensor networks into actionable environmental intelligence. By integrating quantum-hybrid computing with geospatial simulation, this study proposes a method to enhance the computational processing of sensor data, thereby improving the accuracy and reliability of urban monitoring systems.

The manuscript proposes a comprehensive framework that serves as a foundational step toward this vision, integrating advanced computational techniques to enhance forecasting and modeling capabilities for municipal-level environmental monitoring.

The field of environmental forecasting has evolved significantly, moving from first-principle models to data-driven approaches. Traditionally, Chemistry-Transport Models (CTMs) have been the cornerstone of air quality prediction. These models numerically solve complex differential equations describing the physical and chemical processes in the atmosphere [1]. While powerful, CTMs are computationally expensive and their accuracy is often limited by uncertainties in the underlying emissions inventories and meteorological inputs [1].

To overcome these limitations, statistical and machine learning models have gained prominence. Classical time-series methods, such as the AutoRegressive Integrated Moving Average (ARIMA) model, have been widely used for their simplicity and interpretability in capturing linear dependencies and seasonal patterns in environmental data [5,6]. However, they often struggle with the strong non-linearities inherent in environmental systems [1].

The advent of deep learning has revolutionized data-driven environmental science [7,8]. Recurrent Neural Networks (RNNs), particularly Long Short-Term Memory (LSTM) and Gated Recurrent Unit (GRU) architectures, are adept at modeling temporal dependencies and are widely used for forecasting time-series data like dissolved oxygen levels or drought indices [2]. Convolutional Neural Networks (CNNs), traditionally used for image analysis, have been adapted to extract spatiotemporal features from gridded environmental data [7,8]. More recently, the Transformer architecture, with its self-attention mechanism, has shown exceptional capability in capturing long-range dependencies in sequential data, making it a powerful tool for tasks like climate prediction and long-term drought forecasting [9,10].

The next frontier in computational science is Quantum Machine Learning (QML), an emerging field that seeks to leverage the principles of quantum mechanics to solve complex computational problems [11,12]. Quantum computing, through phenomena like superposition and entanglement, offers a fundamentally different way to process information, with the theoretical potential to model the intricate correlations and high-dimensional state spaces characteristic of environmental systems more efficiently than classical computers [13]. Current research in applied QML focuses on hybrid quantum-classical models, where a classical neural network performs initial feature processing, and a parameterized or Variational Quantum Circuit (VQC) acts as a specialized co-processor [14].

Recent research highlights a significant shift from theoretical QML to applied environmental modeling.

Thus, research [15] demonstrated a quantum machine learning approach to spatiotemporal emission modeling, comparing a quantum quanvolutional neural network against a classical ConvLSTM. These findings indicated that quantum models could achieve lower loss and higher accuracy in emission concentration forecasts, suggesting that quantum layers can effectively capture complex spatial features in environmental satellite data. Similarly, in the agricultural domain, work [16] proposed a hybrid quantum deep learning model combining Bi-LSTM with quantum feature processing for yield forecasting. Their results showed that quantum circuits could explore high-dimensional feature spaces more efficiently than classical methods, significantly improving prediction accuracy (R^2^ ≈ 0.99).

The broader applicability of “quantum-like” data modeling in applied sciences has also been reviewed in [17], arguing that quantum probability frameworks can better model uncertainty and complex system behaviors than traditional approaches. Furthermore, research [18] presents successful application of Hybrid Quantum-Classical Neural Networks (H-QNN) to image classification tasks, outperforming classical CNNs with fewer parameters, which supports the hypothesis that quantum-hybrid architectures can offer computational efficiency advantages in processing high-dimensional sensor data.

These hybrid approaches are being actively explored for various tasks, including financial and scientific time-series forecasting, demonstrating the potential to achieve comparable or superior performance with fewer parameters.

Despite these advancements, several critical gaps remain in the application of advanced computational methods to urban environmental monitoring. First, the existing research often treats forecasting, spatial analysis, and simulation as isolated disciplines. There is a clear need for a unified, conceptual framework that integrates these components into a cohesive analytical pipeline, especially one that can accommodate emerging technologies like QML and Large Language Model (LLM)-based analysis. The fragmented presentation of these elements in many studies hinders the development of a holistic environmental intelligence system [2].

Second, while QML holds significant theoretical promise, its practical application to real-world problems is still in its infancy. Comprehensive, large-scale empirical studies that benchmark hybrid quantum models against a wide array of state-of-the-art classical models are rare. In particular, their performance on noisy, limited, and non-stationary environmental sensor data–the kind typically encountered in practical deployments–is not well understood. The field requires rigorous empirical validation to move beyond theoretical promise and assess the true utility of near-term quantum devices [11].

## 2. Analysis of the State-of-the-Art in the Field

Many current studies examine the effectiveness of using quantum neural networks for time series forecasting in the financial sector.

The authors in [19] investigate the use of parameterized quantum circuits (PQCs) as quantum neural networks for time series forecasting. They compare the performance of PQCs with classical bidirectional long short-term memory networks and find that quantum models can achieve similar or better accuracy with fewer parameters and faster training.

The study [20] considers the application of one-dimensional quantum convolutional neural networks (QCNNs) for time series prediction. The use of Fourier transform allows for control over the design of QCNNs, which improves their performance. The authors demonstrate that even with a limited number of parameters, quantum circuits are able to effectively model complex functions, which reduce training time.

In [21], the Quantum Gramian Angular Field method was proposed, which combines quantum computing with deep learning for financial time series forecasting. The research involves transforming stock return data into two-dimensional images using specially designed quantum circuits, allowing convolutional neural networks (CNNs) to be used for forecasting. The results show significant improvements in accuracy compared to classical methods.

The authors in [22] propose a method for learning temporal data using parameterized quantum circuits that have a structure similar to recurrent neural networks. In this model, some of the qubits store past data, while others are used to predict and encode new input data. The study demonstrates the ability of quantum circuits to effectively learn and predict time series.

Quantum computing is also actively used for the analysis of environmental parameters and forecasting, allowing us to compare their efficiency with classical algorithms.

One such study [23] proposes the integration of quantum computing with the Internet of Vehicles (IoV) for environmental monitoring and rapid response to environmental threats. Using quantum sensors installed on vehicles, the system provides accurate air quality assessment. Proposed Quantum Mesh Network Fabric dynamically adjusts the quantum network topology according to traffic, maintaining the integrity of quantum states and ensuring reliable data transmission. Additionally, the use of a variational quantum classifier and quantum entanglement techniques allows for reduced delays in the transmission of danger signals, which contributes to the prompt informing of emergency services and the population.

Another study [24] focuses on the use of quantum algorithms for stock price prediction. The authors conduct a series of experimental simulations using both classical and quantum hardware. They use quantum annealing for feature selection and principal component analysis to reduce the dimensionality of the data. The prediction problem is transformed into a classification problem, where a quantum support vector machine is used to predict price movements (up or down). The results are compared with classical models by analyzing the accuracy of the predictions and the F-measure.

Separately, deep neural networks, such as LSTM, GRU, CNN, and Transformer, are actively used to predict environmental indicators.

One study [25] compares the performance of different deep learning models, including CNN, TCN, LSTM, GRU, and BiRNN, for multi-step prediction of dissolved oxygen levels in water. Using data from the Yangtze River from 2012 to 2016, the authors found that the GRU model performed best in terms of RMSE, MAE, and coefficient of determination, highlighting its effectiveness in predicting ecological parameters.

Another study [26] focused on long-term drought assessment based on geospatial satellite data. The authors first employ an LSTM model (three layers, 256 hidden cells) to fill gaps in satellite soil moisture data, using inputs like temperature, precipitation, ET, and runoff. Subsequently, basin-specific LSTM models (also three layers, 256 hidden cells) are developed to forecast soil moisture. The most promising output of this work is the integration of the LSTM models with an interpretable AI method called Expected Gradients (EG), which allows the framework to quantify the specific contribution of each input feature (such as precipitation or temperature) to the soil moisture predictions over time, thereby providing a crucial understanding of the mechanisms driving drought development.

However, classical architectures continue to evolve. Recent studies have proposed sophisticated hybrid classical models to address non-linearity in sensor data. For instance, work [27] presents developed an IoT-based monitoring system utilizing a hybrid LSTM-GRU model for real-time power forecasting. This work demonstrated that combining different recurrent architectures can mitigate the specific limitations of standalone LSTM or GRU models, achieving superior accuracy in real-time IoT scenarios. In the financial domain, which shares time-series characteristics with environmental data, work [28] introduces a decomposition-ensemble model (CEEMDAN-SE combined with ARIMA-CNN-LSTM). This approach effectively handled non-stationarity by decomposing complex signals into high- and low-frequency components, a methodology relevant to processing volatile environmental sensor readings.

Some of the studies analyze the use of mathematical modeling methods for pollution assessment based on spatial interpolation, in particular using the inverse weighted distance method, for the analysis of environmental data and time series of changes in environmental parameters.

Work [29] presents a course on environmental geographic information systems (GIS), which includes an overview of the concepts and methods used to analyze and model environmental data. The course structure and examples of its practical use for solving environmental problems are presented.

The paper [30] is devoted to the use of remote sensing and open source software for working with GIS in ecology. The authors describe approaches to spatial data analysis, including tools for creating pollution maps and monitoring the state of the environment.

The source [31] discusses the basics of using GIS for environmental modeling and engineering. It describes methods of spatial analysis, interpolation, and prediction of environmental parameters. It provides examples of creating thematic maps and developing models for planning environmental protection measures.

Some basic research is devoted to computer modeling of changes in pollutant concentrations in local areas, which allows for effective assessment of the impact of man-made sources on the environment.

In [32], the authors propose a new parameterization of the concentration flux using fractional calculus for modeling the dispersion of pollutants within the planetary boundary layer of the atmosphere. The mathematical approach and its application to environmental monitoring problems are discussed in detail.

The paper [33] is devoted to the atmospheric transport and diffusion model HYSPLIT, developed by NOAA. The authors describe the functional capabilities of the model, its application to assess the transport and dispersion of pollutants in the air, as well as examples of practical use in real conditions.

Reference [34] examines atmospheric observations and methods for evaluating atmospheric chemical composition models. It describes the importance of modeling for the analysis of atmospheric processes, discusses methods for model validation, and examples of their application in predicting environmental changes.

Regarding computational simulation frameworks, work [35] presents HybriD-GM, a framework for quantum computing simulation on hybrid parallel architectures. While this work focuses on the hardware-level optimization of quantum simulations (CPU/GPU integration), it highlights the computational constraints inherent in simulating quantum circuits for large-scale applications. This underscores the necessity for frameworks like the proposed DPPDMext, which are designed to abstract these complexities and integrate simulation outputs directly into the decision-making pipeline of environmental monitoring systems.

Currently, there is limited research that directly combines the use of Vision models and large language models to analyze graphs and numerical metrics for data quality assessment. However, several works have been taken as a basis for synthesizing the concept of neural network agents for prediction evaluation and modeling tasks.

In [36], the use of deep neural networks based on LSTM autoencoders for predicting the transition of barley genotype to phenotype is considered. The authors describe in detail the architecture of the model and its effectiveness in modeling the relationship between genetic and phenotypic characteristics.

Work [37] devoted to the basics of mining concepts and techniques. The authors consider key data analysis methods, in particular clustering, associative rules and classification, and also demonstrate their practical application in solving a wide range of problems in various industries.

In existing studies, a number of systems and methods for collecting, processing and analyzing data on environmental pollution have been developed. These works are aimed at increasing the efficiency of environmental monitoring and creating a basis for making management decisions. Currently, technologies for predicting environmental data are being studied, which allows improving the quality of measures for regulating policies in the field of combating environmental pollution.

In [38], an information and analytical system for collecting, processing and analyzing air pollution data is presented. The system architecture, methods for automating data collection and algorithms for data processing aimed to assess the atmosphere conditions re described. Special attention is paid to the integration of technological and business processes within the system.

The paper [39] is devoted to the development of an information system for collecting and storing air quality data at the municipal level using VAISALA stations. The technical aspects of the system implementation, data collection algorithms, and practical possibilities for air monitoring are considered.

The study [40] describes a method for automatically generating reports on the number of exceedances of established standards for atmospheric markers. A data processing algorithm is proposed for operational air quality control and presentation of results in the form of reports that can be used in environmental monitoring.

In the paper [41], a web-based technology for intelligent analysis of environmental data at the industrial enterprise is presented. The applied methods of data collection, processing and analysis using modern web-oriented tools are described. Examples of practical use of the developed system for monitoring the environmental conditions and making decisions on reducing the man-made impact on the environment are given.

## 3. A Unified Framework for Environmental Data Analysis

### 3.1. Current Research Gaps

Despite the recent achievements in the field, analyzed in the previous chapter, several critical gaps remain in the application of advanced computational methods to urban environmental monitoring. First, the existing sources often treat forecasting, spatial analysis, and simulation as isolated disciplines. There is a clear need for a unified, conceptual framework that integrates these components into a unified analytical pipeline, especially one that can accommodate emerging technologies like QML and Large Language Model (LLM)-based analysis. The fragmented presentation of these elements in many studies prevents the development of a holistic environmental intelligence system.

Second, while QML holds significant theoretical promise, its practical application to real-world problems is still at the initial stage. Comprehensive, large-scale empirical studies that benchmark hybrid quantum models against a wide array of state-of-the-art classical models are rare. In particular, their performance on noisy, limited, and non-stationary environmental sensor data—the kind typically encountered in practical deployments—is not well understood. The field requires rigorous empirical validation to move beyond theoretical promise and assess the true utility of near-term quantum devices [11].

This manuscript addresses these gaps through four primary contributions:

1. The proposal of DPPDMext, a novel conceptual framework for advanced environmental data analytics that formally integrates forecasting, geospatial and simulation modeling, and AI-driven expert evaluation into a single, extensible system.
2. A large-scale, systematic benchmark of thirteen classical and quantum-hybrid forecasting models applied to real-world municipal environmental sensor data. This provides critical insights into the practical performance of QML under data-scarce and noisy conditions, representing one of the most extensive comparative studies of its kind in the environmental domain.
3. The development of an integrated methodology combining spatial interpolation for creating virtual sensor stations and an A* algorithm-based traffic simulator for generating and testing hypotheses about dynamic pollution sources.
4. A proof-of-concept for using a Vision-Language Model (VLM) agent to automate the preliminary analysis and interpretation of complex scientific results, demonstrating a path toward accelerating the research workflow in computational environmental science.

### 3.2. The DPPDMext Conceptual Model

The concept of preparing and processing data for decision-making (Data Preparation and Processing for Decision-Making) in an ecological information system can be formalized as a set:

DPPDM = (D, T, P, F, V),

where D (Data)—a set of environmental data obtained from reliable sources (for example, from Vaisala automatic stations, stationary observation points, etc.), including primary measurements of pollutant concentrations, temperature, humidity, etc.; T (Transformation)—a set of operations for normalization, interpolation, filling in missing data and aggregation of indicators to improve the quality of the analysis; P (Prediction)—the use of forecasting methods (LSTM, GRU, Transformer) to estimate future changes in environmental parameters; F (Feature Engineering)—a set of additional indicators that are calculated based on primary data, for example, maximum permissible concentrations (MPC), air pollution indices, statistical characteristics of time series, etc.; V (Visualization)—presentation of results in the form of time series graphs, pollution maps and other analytical visualizations.

In an expanded form, the preparation, data processing, and augmented forecast can be represented as:

Pext = (A, P, Q, M, E),

DPPDMext = (D, T, F, P ext, F, V),

DPPDMext = (D, T, F, (A, P, Q, M, E), F, V),

where A (Analysis)—analysis of time series of environmental parameters for expert determination of possible best forecasting or modeling strategies; Q (Quality optimization)—application of deep ensemble methods or automated optimization of the choice of forecasting models; M (Modeling)—modeling of a local area of the studied area using simulators or creation of virtual forecasting stations to predict different states of the system; E (Expert)—expert evaluation of forecasting and modeling models using forecasting quality metrics, to determine the forecasts of parameters on the basis of which additional indicators can be selected.

This concept describes not only classical data processing, but also their advanced integration and analysis within environmental information systems.

### 3.3. Integration of Geospatial and Simulation Modules into Forecasting

An innovation of the DPPDMext framework is the tight coupling between the geospatial analysis (M) and the time-series forecasting (P) modules, rather than treating them as isolated tasks. The output of the geospatial interpolation (IDW) and the pollution simulator is not merely for visualization; it serves as a feature engineering mechanism (F). Specifically, the “background” pollution levels ($C_{background}$) derived from the IDW analysis and the dynamic traffic density indices generated by the A* simulator are injected as exogenous variables (external regressors) into the classical layers of the hybrid forecasting models. This allows the LSTM or Quantum-LSTM architectures to learn correlations not only from historical temporal lags but also from spatial dynamics, effectively transforming the problem from univariate to multivariate time-series forecasting.

### 3.4. System Architecture

**Figure 1:**
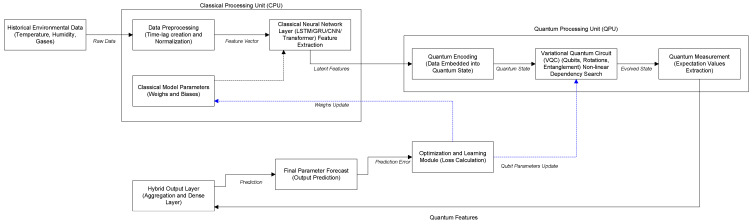
Generalized architecture of the hybrid quantum-classical forecasting model illustrating the interaction between CPU and QPU processing units.

As depicted in Figure 1, the proposed architecture synergizes classical and quantum computing paradigms. A Classical Processing Unit (CPU) is responsible for the initial data preprocessing and feature extraction, utilizing deep learning layers such as LSTM, GRU, or Transformers. The extracted features are then mapped into a quantum state within the Quantum Processing Unit (QPU). Here, a Variational Quantum Circuit (VQC) is employed to process the latent feature space, capturing complex non-linear dependencies that are often intractable for classical algorithms alone. The quantum measurement results are subsequently aggregated in a hybrid output layer to generate the final forecast. The entire system is trained end-to-end, with an optimization module employing a feedback loop to simultaneously update both classical weights and quantum circuit parameters based on the prediction error.

**Figure 2:**
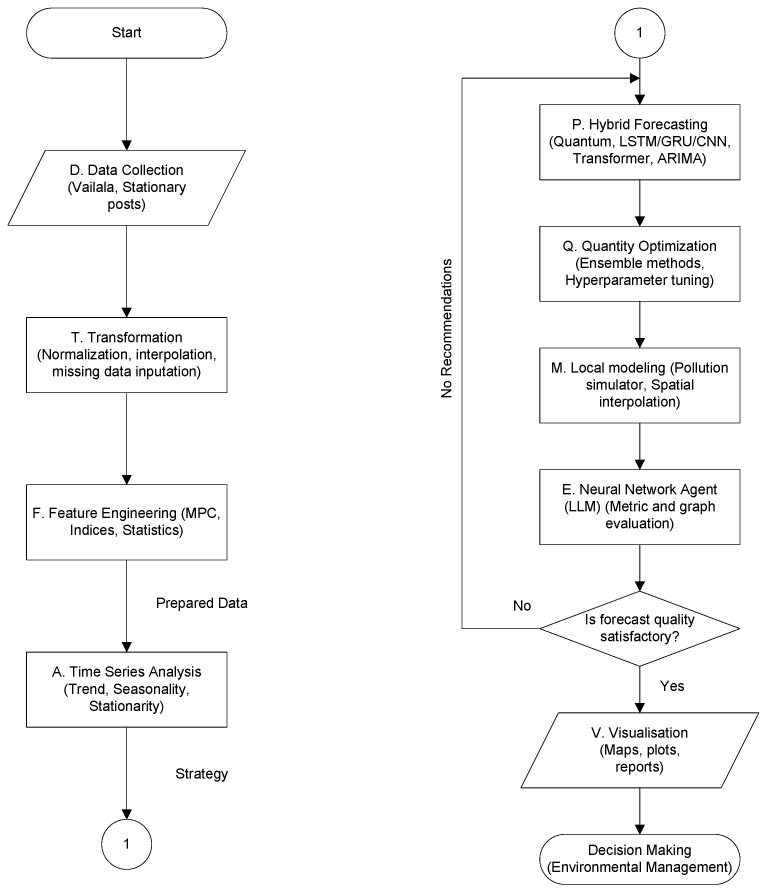
Logical flowchart of the DPPDMext pipeline.

The operational logic of the framework is formalized in the Extended Data Preparation and Processing for Decision-Making (DPPDMext) pipeline, shown in Figure 2. The workflow initiates with the data acquisition (D), followed by rigorous transformation (T) and feature engineering (F) to prepare the inputs. The core innovation lies in the extended prediction phase ($P_{ext}$), which integrates four sequential stages: preliminary time series analysis (A), the execution of hybrid quantum-classical forecasting models (P), quality optimization (Q), and local environmental simulation (M). A distinct feature of this pipeline is the inclusion of an LLM-based neural network agent (E). This agent evaluates the forecasting outputs; if the quality metrics fall below a defined threshold, the agent triggers a feedback loop, providing recommendations for model refinement. Upon validation, the successful forecasts proceed to the visualization stage (V) to facilitate evidence-based environmental management.

## 4. Materials and Methods

### 4.1. Study Area and Dataset Characteristics

The data for this study were sourced from three automated Vaisala AQT420 environmental monitoring stations (Vaisala, Vantaa, Finland) located in distinct zones within the city of Kremenchuk, Ukraine: “Gymnasium No. 26” (a residential/educational area), the “Northern Industrial Node” (an industrial zone), and the “Favorit” district (a high-traffic commercial area). The dataset comprises monthly averaged readings for five key parameters: relative humidity (%), ambient temperature (°C), atmospheric pressure (GPa), carbon monoxide (CO, mg/m^3^), and nitrogen dioxide (NO_2_, mg/m^3^). The data spans a period of 35 months, providing a limited but challenging time series for forecasting.

From a mathematical point of view, the data can be represented as a set of time series $X_{t},$ for *t* = 1, 2, …, T, where T is the number of observations.

#### 4.1.1. Advanced Data Preprocessing and Transformation

To ensure the robustness of the quantum-hybrid models against sensor noise and irregularities, a rigorous preprocessing pipeline (part of the T component in the DPPDM framework) was implemented.

Minor gaps in the time series (less than 6 h) resulting from temporary sensor outages were handled using linear interpolation to preserve local trends. Larger gaps were addressed by excluding the affected timestamps to prevent the introduction of synthetic artifacts.

Anomalous readings caused by hardware malfunctions were identified using the Z-score method. Data points with a Z-score >3σ (three standard deviations from the rolling mean) were treated as outliers and replaced with the local median value to maintain statistical stability.

All input features were normalized using Min-Max scaling to map values into the [0,1] range. This step is critical for the quantum embedding layers (e.g., AngleEmbedding), as it ensures that input features correspond to valid rotation angles (scaled by π or 2π) within the quantum gates, preventing saturation and ensuring effective gradient descent during training.

For the geospatial component, Inverse Distance Weighting (IDW) was utilized with a smoothing parameter p=2. This method was chosen over Kriging for its computational efficiency in generating continuous pollution surfaces from the sparse set of fixed monitoring stations ($S=\{(x_{i},y_{i},C_{i})\}$), enabling the creation of virtual data points for areas lacking physical sensors.

#### 4.1.2. Mathematical Framework for Time Series Analysis

The average value is defined as:

$$\mu =\frac{1}{T}\sum_{t=1}^{T}X_{t}.$$

The variance for environmental time series is determined by the following relationship:

$$\sigma^{2}=\frac{1}{T-1}\sum_{t=1}^{T}{\left(X_{t}-\mu \right)}^{2},$$

where the standard deviation is:

$$\sigma =\sqrt{\sigma^{2}}.$$

The interquartile range is defined as:

$$IQR=Q_{3}-Q_{1},$$

where $Q_{1}$ and $Q_{3}$ are the first and third quartiles.

Trend analysis can be performed using STL decomposition:

$$X_{t}=T_{t}+S_{t}+R_{t},$$

where $T_{t}$ is the trend, $S_{t}$ is the seasonal component, $R_{t}$ is the residual component.

Two approaches are used to detect seasonality: the autocorrelation function (ACF) and the Fourier transform (FFT).

The autocorrelation for lag *k* is defined as:

$$\mathrm{ACF}\left(k\right)=\frac{\sum_{t=1}^{T-k}\left(X_{t}-\mu \right)\left(X_{t+k}-\mu \right)}{\sum_{t=1}^{T}{\left(X_{t}-\mu \right)}^{2}},$$

where *μ* is the average value of the series.

The FFT of a time series gives the frequency components:

$$\mathrm{FFT}\left(X_{t}\right)=\sum_{n=0}^{T-1}X_{t}e^{-2\pi i\frac{nk}{T}},$$

where $X_{t}$ is the value of the time series, *T* is the number of observations.

Time series can be classified according to three main characteristics: trend, seasonality, and stationarity.

The trend is determined by linear approximation:

$$X_{t}=at+b,$$

where *a* is the slope of the line (trend), *b* is the segment.

Seasonality is detected through autocorrelation, where a series is considered seasonal if several values are observed for ACF values at lags greater than 0.1:

$${\mathrm{ACF}}_{k}>0.1\;\mathrm{for}\;k>0,$$

If this is done, the series is classified as seasonal.

Stationarity is determined through the ADF (Augmented Dickey–Fuller Function) test. A series is considered stationary if the *p*-value is less than 0.05:

p-value<0.05⇒stationary series.

To analyze lag autocorrelation, *k,* the following relationship is used:

$$\mathrm{ACF}\left(k\right)=\frac{\mathrm{Cov}\left(X_{t},X_{t+k}\right)}{\mathrm{Var}\left(X_{t}\right)},\;$$

where $\mathrm{Cov}\left(X_{t},X_{t+k}\right)$ is the covariance between the values of the time series at lag *k*, and $\mathrm{Var}\left(X_{t}\right)$ is the variance of the time series.

Other metrics were determined by algorithmic methods available in the Pandas library (v.2.2.2, NumFOCUS, Austin, TX, USA).

### 4.2. Determining the Indicators of the Input Dataset

A preliminary analysis of the dataset reveals significant heterogeneity across locations and parameters, posing a considerable challenge for predictive modeling. The key statistical properties of the time series are summarized in Table 1. The dataset is characterized by strong seasonality and high volatility in meteorological parameters (temperature and humidity). In contrast, the pollutant series (CO and NO_2_) exhibit strong, increasing trends and are non-stationary, as confirmed by the ADF test *p*-values being well above the 0.05 threshold for stationarity. This non-stationarity indicates underlying systemic changes in pollution levels over the study period. Furthermore, high lag-1 autocorrelation values for most parameters suggest strong temporal dependence, a feature that time-series models are designed to exploit. The combination of non-stationarity, seasonality, high variance, and a short data record (less than three full seasonal cycles) makes this a rigorous test case for evaluating the robustness and adaptability of advanced forecasting models.

Table 1 consolidates descriptive statistics for the time-series data from three monitoring locations. Trend is the slope of a linear regression. Seasonality is the lag with the highest ACF value. Stationarity is determined by the ADF test (*p* < 0.05 indicates stationarity). Autocorrelation is the Lag-1 ACF value.

The determination of central tendencies for Gymnasium No. 26 indicates a certain stability of climatic and environmental parameters. The average temperature is 11.84 °C, which indicates a cool climate. The average humidity is 70.03%, which emphasizes the relatively high humidity compared to other areas of the city. The mode indicates the lowest values of temperature (−1.71 °C) and humidity (49.18%), which indicates possible extreme weather conditions, but these values are less typical compared to the median, which has values close to the average.

The Northern Industrial Node, with an average temperature of 15.90 °C, has a warmer climate. Humidity is significantly lower here than in Gymnasium No. 26, at 62.46%. Compared to other areas, this area is characterized by higher levels of carbon monoxide and nitrogen dioxide, which is evidence of intensive industrialization and the impact of industrial facilities. The trend for this area also indicates temperatures that are much lower than average, indicating the possibility of extreme weather conditions with low temperatures (−1.67 °C). At the same time, the median temperature (18.67 °C) is significantly higher than average, highlighting the hotter conditions in this area.

The Favorite district has the highest average temperature (16.92 °C) and the lowest humidity levels (54.88%) compared to other districts. This may indicate a drier climate. Carbon monoxide and nitrogen dioxide levels are also at high levels, which is associated with traffic congestion and air pollution in this densely populated area. The mode for temperature indicates even lower values (−2.53 °C), which demonstrates possible strong fluctuations in weather conditions within this district. The median, as for the Northern Industrial Zone, indicates higher values, which emphasizes the increased stability of temperature conditions compared to other areas of the city.

### 4.3. Forecasting Models: Classical and Quantum-Hybrid Architectures

A comprehensive suite of thirteen forecasting models was implemented to evaluate a wide spectrum of computational approaches. These models can be broadly categorized into statistical methods, classical deep learning architectures, and their corresponding quantum-hybrid versions.

#### 4.3.1. General Quantum-Hybrid Methodology

The quantum-hybrid models developed in this study follow a common architectural pattern that integrates classical deep learning with a VQC. This approach is representative of current strategies for applying near-term quantum computers to machine learning tasks [12,13,14]. The general process is as follows:

1. A classical model (e.g., an LSTM or CNN) processes the input time series to extract relevant temporal or spatial features, producing a classical feature vector.
2. This classical vector is then encoded into the state of a quantum system using a data-embedding layer, such as AngleEmbedding, which maps classical data values to the rotation angles of quantum gates [2].
3. The encoded quantum state is processed by a VQC, which is a quantum circuit composed of parameterized quantum gates (e.g., single-qubit rotations) and fixed entangling gates (e.g., CNOTs). The parameters of the rotation gates are trainable, analogous to the weights in a classical neural network [42].
4. Finally, a measurement is performed on the qubits. The expectation values of these measurements (e.g., with respect to the Pauli-Z operator) form the output of the quantum layer, which is then used to produce the final forecast. The entire hybrid system is trained end-to-end using classical optimization algorithms to minimize a loss function [43].

#### 4.3.2. Recurrent Architectures

(a) LSTM (Long Short-Term Memory). A type of RNN that uses gating mechanisms (input, forget, output gates) to control the flow of information, enabling it to learn long-term dependencies in sequential data [44,45].

The basic LSTM model is formalized as follows:

$$h_{t}=\sigma \left(W_{h}\cdot x_{t}+U_{h}\cdot h_{t-1}+b_{h}\right),$$

where $h_{t}$ is the LSTM state at time *t*, $x_{t}$ is the input feature vector, $W_{h}$, $U_{h}$ are weight matrices, $b_{h}$ is the shift vector, *σ* is the activation function.

The forecasting process includes following steps.

1. Creating lag features:

$$X_{\mathrm{lag}}=\left[\begin{array}{ccc} y_{t-\mathrm{lag}}, & \ldots , & y_{t-1} \end{array}\right],$$

where *lag* is the number of previous time points taken into account.

2. The input data is converted into tensor format for use in the LSTM.

3. The model is optimized using the loss function:

$$L=\frac{1}{N}\sum_{i=1}^{N}{\left(y_{i}-{\hat{y}}_{i}\right)}^{2},$$

where *N* is the number of samples, $y_{i}$ is the true value, ${\hat{y}}_{i}$ is the predicted value.

A quantum LSTM prediction model involves the use of quantum computing to improve prediction.

The main stages include following steps.

1. The quantum scheme is implemented using data embedding methods:

“AngleEmbedding” (x) ”and” ”BasicEntanglerLayers” (w),

where *x* is the feature vector, *w* is the quantum scheme weights.

2. The output values are read as mathematical expectations of the Pauli-Z operators:

$$\langle Z\rangle_{i}=\mathrm{Tr}\left(\rho \cdot Z_{i}\right),$$

where *ρ* is the state of the quantum system, $Z_{i}$ is the Pauli-Z operator for the *i*-th qubit.

3. The output of the quantum model is aggregated to form a prediction:

$$\hat{y}=\frac{1}{Q}\sum_{i=1}^{Q}\langle Z\rangle_{i},$$

where *Q* is the number of qubits.

(b) GRU (Gated Recurrent Unit). A simplified version of the LSTM with fewer parameters, using update and reset gates. It often achieves comparable performance with greater computational efficiency.

A prediction method based on recurrent neural networks using GRU, which has the following equation:

$$h_{t}=\left(1-z_{t}\right)⊙h_{t-1}+z_{t}⊙{\tilde{h}}_{t},$$

where $h_{t}$ is the GRU state at time *t*, $z_{t}$ is the updating gateway, ${\tilde{h}}_{t}$ is the candidate state, ⊙ is the element-wise multiplication. The updating gateway is defined as:

$$z_{t}=\sigma \left(W_{z}x_{t}+U_{z}h_{t-1}\right),$$

and the candidate state:

$${\tilde{h}}_{t}=tanh\left(W_{h}x_{t}+U_{h}\left(r_{t}⊙h_{t-1}\right)\right),$$

where $r_{t}$ is the discharge gate.

The forecasting process includes following steps.

1. Input data in the form of lag features are formed as:

$$X_{\mathrm{lag}}=\left[\begin{array}{ccc} y_{t-\mathrm{lag}}, & \ldots , & y_{t-1} \end{array}\right].$$

2. The training loss function is defined as:

$$L=\frac{1}{N}\sum_{i=1}^{N}{\left(y_{i}-{\hat{y}}_{i}\right)}^{2},$$

where *N* is the number of examples, $y_{i}$ is the true value, ${\hat{y}}_{i}$ is the predicted value.

3. During prediction, the output values ${\hat{y}}_{t}$ are formed based on the state of the GRU.

The quantum prediction method combines GRU with quantum computing. The main steps include the following.

1. For quantum computing, a quantum scheme is used:

$$\mathrm{circuit}\left(x,w\right)=\left[\left\langle Z_{1}\rangle ,\ldots ,\langle Z_{Q}\right\rangle \right],$$

where *x* is the input data, *w* is the parameters of the quantum circuit, *Q* is the number of qubits.

2. The output of the quantum circuit is calculated as:

$$\left\langle Z_{i}\right\rangle =\mathrm{Tr}\left(\rho \cdot Z_{i}\right).$$

3. The output of the GRU is used as input to the quantum circuit. The prediction is formed as:

$$\hat{y}=\frac{1}{Q}\sum_{i=1}^{Q}\langle Z_{i}\rangle ,$$

which aggregates the results of quantum calculations.

GRU allows for modeling time series with non-linear dependencies. The quantum approach complements GRU by providing the ability to handle complex correlations using quantum superposition and entanglement.

#### 4.3.3. Attention-Based Architecture (Transformer)

This architecture relies entirely on a self-attention mechanism to weigh the importance of different parts of the input sequence, allowing it to model dependencies regardless of their distance [46]. It includes the following steps.

1. Lag features for time series are formed as follows:

$$X_{\mathrm{lag}}=\left[\begin{array}{ccc} y_{t-\mathrm{lag}}, & \ldots , & y_{t-1} \end{array}\right].$$

2. Multi-headed self-attention is calculated by the formula:

$$\mathrm{Attention}\left(Q,K,V\right)=\mathrm{softmax}\left(\frac{QK^{⊤}}{\sqrt{d_{k}}}\right)V,$$

where *Q*, *K*, *V* are the matrices of queries, keys, and values, respectively, and $d_{k}$ is the dimension of the keys.

3. After calculating the self-attention weights, the data is passed through layer normalization and a dense layer:

$${\hat{y}}_{t}=W_{d}z_{t}+b_{d},$$

where $W_{d}$ and $b_{d}$ are the parameters of the dense layer.

The quantum method complements the transformer architecture with a quantum layer for processing lag features.

1. Lag features $X_{\mathrm{lag}}$ are fed to the input of the quantum layer, which implements the quantum scheme:

$$\mathrm{QuantumCircuit}\left(X_{\mathrm{lag}}\right)=\left[\left\langle Z_{1}\rangle ,\ldots ,\langle Z_{Q}\right\rangle \right],$$

where *Q* is the number of qubits, $\left\langle Z_{i}\right\rangle$ is the expected value of the Pauli operator *Z* on the *i*-th qubit.

2. For quantum computing, a parameterized scheme is used:

$$\left\langle Z_{i}\right\rangle =\mathrm{Tr}\left(\rho \cdot Z_{i}\right).$$

3. The output of the quantum circuit is integrated into the transformer architecture through a dense layer:

$${\hat{y}}_{t}=W_{q}z_{q}+b_{q},$$

where $z_{q}$ is the vector of quantum features, $W_{q}$ and $b_{q}$ are the parameters of the dense layer.

The non-quantum method is effective for solving forecasting problems, using self-attention mechanisms to analyze dependencies in time series.

The quantum method additionally takes into account correlations using quantum computing, which can improve prediction accuracy.

#### 4.3.4. Convolutional and Feed-Forward Architectures (CNN, MLP)

(a) CNN (Convolutional Neural Network). A 1D CNN is used to extract local patterns and features from the time series by sliding a learned kernel across the input sequence [47].

Prediction using a convolutional neural network (CNN) has the following main steps.

1. Input data is converted into lag features:

$$X_{\mathrm{lag}}=\left[\begin{array}{ccc} y_{t-\mathrm{lag}}, & \ldots , & y_{t-1} \end{array}\right].$$

2. The convolutional layer performs the operation:

$$Z_{t}=\sigma \left(W\times X_{\mathrm{lag}}+b\right),$$

where *W* is the convolution kernel, *b* is the bias, *σ* is the activation function (for example, ReLU), × is the convolution operation.

3. After the convolutional layer, a smoothing layer (Flatten) is used, followed by a dense layer (Dense), which predicts the original value:

$${\hat{y}}_{t}=W_{d}z_{t}+b_{d},$$

where $W_{d}$ and $b_{d}$ are the parameters of the dense layer.

4. The loss function is defined as the mean square error (MSE):

$$L=\frac{1}{N}\sum_{i=1}^{N}{\left(y_{i}-{\hat{y}}_{i}\right)}^{2},$$

where *N* is the number of samples, $y_{i}$ is the actual value, ${\hat{y}}_{i}$ is the predicted value.

The quantum prediction method combines CNN with quantum computing.

1. The output of the convolutional network $z_{t}$ is used as the input of the quantum model. The quantum circuit performs the following operations:

$$\mathrm{circuit}\left(z_{t},w\right)=\left[\left\langle Z_{1}\rangle ,\ldots ,\langle Z_{Q}\right\rangle \right],$$

where $z_{t},w$ are the parameters of the quantum scheme, *Q* is the number of qubits, $\left\langle Z_{i}\right\rangle$ is the expected value of the Pauli operator *Z* on the *i*-th qubit.

2. For quantum computing, the parameterization technique is used:

$$\left\langle Z_{i}\right\rangle =\mathrm{Tr}\left(\rho \cdot Z_{i}\right).$$

3. The prediction is formed as the average value of the outputs of the quantum circuit:

$${\hat{y}}_{t}=\frac{1}{Q}\sum_{i=1}^{Q}\langle Z_{i}\rangle .$$

The non-quantum approach is based on classical convolutional networks, which effectively extract local spatiotemporal features.

The quantum approach allows for the consideration of complex correlations between data through quantum superposition and entanglement, which can improve prediction.

(b) MLP (Multilayer Perceptron). A basic feed-forward neural network that learns a mapping from a window of lagged input values to the forecast value.

The basic method is based on the use of a multilayer perceptron.

1. To construct lag features, a time series vector is used:

$$X_{\mathrm{lag}}=\left[\begin{array}{ccc} y_{t-\mathrm{lag}}, & \ldots , & y_{t-1} \end{array}\right].$$

2. The MLP model consists of $n_{\mathrm{layers}}$ layers. Each layer is represented by a non-linear activation function:

$$z^{\left(l\right)}=f\left(W^{\left(l\right)}z^{\left(l-1\right)}+b^{\left(l\right)}\right),$$

where $W^{\left(l\right)}$ and $b^{\left(l\right)}$ are the weights and biases of the l-th layer, *f*(⋅) and is the activation function (for example, ReLU).

3. The last layer of the model calculates the forecast:

$${\hat{y}}_{t}=W_{\mathrm{out}}z^{\left(n_{\mathrm{layers}}\right)}+b_{\mathrm{out}}.$$

The quantum method uses quantum computing to enrich lag features.

1. Lag features are formed similarly to the non-quantum method:

$$X_{\mathrm{lag}}=\left[\begin{array}{ccc} y_{t-\mathrm{lag}}, & \ldots , & y_{t-1} \end{array}\right].$$

2. The quantum layer is based on quantum schemes. For each vector lag features, a parameterized quantum scheme is used:

$$\mathrm{QuantumLayer}\left(x\right)=\left[\left\langle Z_{1}\rangle ,\ldots ,\langle Z_{Q}\right\rangle \right],$$

where *Q* is the number of qubits, $\left\langle Z_{i}\right\rangle$ is the expected value of the Pauli operator Z on the *i*-th qubit.

3. The output of the quantum layer is integrated with the classical MLP model:

$${\hat{y}}_{t}=W_{\mathrm{quant}}z_{\mathrm{quant}}+b_{\mathrm{quant}},$$

where $z_{\mathrm{quant}}$ is the vector of quantum features, $W_{\mathrm{quant}}$ and $b_{\mathrm{quant}}$ is the model parameters.

The non-quantum method is a basic approach to time series modeling using lag features.

The quantum method complements the process of modeling lag features by taking into account additional dependencies using quantum computing.

#### 4.3.5. Statistical and State-Space Models

(a) AutoARIMA. Automatically selects the optimal parameters (p, d, q) for an ARIMA model, which captures dependencies based on past values (AR), differencing for stationarity (I), and past forecast errors (MA) [4].

The quantized AutoARIMA model uses quantum schemes to integrate variational quantum layers and automatically select parameters.

1. For each element of the input vector $x_{initial}$ encoding is performed using rotation “RX”:

$$qml.RX\left(x_{i},\mathrm{wires}=i\right),\;i=1,\ldots ,n_{\mathrm{qubits}},$$

where $n_{\mathrm{qubits}}$ is the number of qubits.

2. Each variational layer consists of a rotation RY for each qubit and quantum couplings CNOT between qubits:

$$qml.RY\left(\theta_{l,i},\mathrm{wires}=i\right),\;l=1,\ldots ,L,\;i=1,\ldots ,n_{\mathrm{qubits}},$$

$$qml.CNOT\left(\mathrm{wires}=\left(i,j\right)\right),\;\left(i,j\right)\in \mathrm{connections}.$$

3. At the output of each qubit, a measurement is performed by the Pauli operator *Z*:

$$\langle Z_{i}\rangle =qml.expval\left(qml.PauliZ\left(i\right)\right),\;i=1,\ldots ,n_{\mathrm{qubits}}.$$

4. The loss function includes the mean square error (MSE) between the true and predicted values, as well as a regularization term to avoid overfitting:

$$L\left(w\right)=\frac{1}{N}\sum_{i=1}^{N}{\left(y_{i}-{\hat{y}}_{i}\right)}^{2}+\lambda ∥w{∥}^{2},$$

where w are the model parameters, λ is the regularization coefficient, N is the number of points in the training sample.

5. Prediction is performed iteratively, using the last value as input to the scheme:

$${\hat{y}}_{t+k}=\mathrm{QuantumLayer}\left({\hat{y}}_{t+k-1},w\right),\;k=1,\ldots ,H,$$

where *H* is the forecasting horizon.

The quantum forecasting model uses variational quantum schemes to account for non-linear dependencies in time series. The integration of quantum computing with classical algorithms allows for high accuracy and unique data processing.

(b) AutoETS. Automatically selects the best exponential smoothing state-space model based on Error, Trend, and Seasonality components.

The AutoETS quantum prediction method can be represented as follows.

1. The seasonal component $y_{\mathrm{seasonal}}$ is calculated as a shift by *seasonal_lag*:

$$y_{\mathrm{seasonal}}\left[t\right]=\left\{\begin{array}{ll} y\left[t-\mathrm{seasonal}\_\mathrm{lag}\right], & t\geq \mathrm{seasonal}\_\mathrm{lag},\\ 0, & t<\mathrm{seasonal}\_\mathrm{lag}. \end{array}\right.$$

2. Adjusted data:

$$y_{\mathrm{adjusted}}\left[t\right]=y\left[t\right]-y_{\mathrm{seasonal}}\left[t\right].$$

3. The matrix of lag variables $X_{\mathrm{lagged}}\in R^{\left(N-\mathrm{lag}\right)\times \mathrm{lag}}$ is defined as:

$$X_{\mathrm{lagged}}\left[i,j\right]=y_{\mathrm{adjusted}}\left[i+j\right],\;i=1,\ldots ,N-\mathrm{lag},\;j=1,\ldots ,\mathrm{lag}.$$

If $\mathrm{lag}>n_{\mathrm{qubits}}$, only the first $n_{\mathrm{qubits}}$ variables are used.

4. The quantum scheme models the relationship between lag variables and prediction:

- Encoding input data into quantum states:

$$qml.RX\left(x_{i},\mathrm{wires}=i\right),\;i=1,\ldots ,n_{\mathrm{qubits}}.$$

- Parameterized rotations for each qubit:

$$qml.RY\left(\theta_{i},\mathrm{wires}=i\right),\;i=1,\ldots ,n_{\mathrm{qubits}}.$$

- Measuring the expectation value of the Pauli operator *Z*:

$$\langle Z_{i}\rangle =qml.expval\left(qml.PauliZ\left(i\right)\right).$$

5. The loss function is defined as the mean square error:

$$L\left(\theta \right)=\frac{1}{N}\sum_{i=1}^{N}{\left({\hat{y}}_{i}-y_{i}\right)}^{2},$$

where ${\hat{y}}_{i}$ is the predicted value, $y_{i}$ is the actual value.

6. Optimization of parameters $\theta_{i}$ is carried out using the Adam algorithm:

$$\theta =\theta -\eta \cdot \nabla_{\theta }L\left(\theta \right),$$

where *η* is the learning rate.

7. Predicting the value $y_{t+1}$ is performed recursively:

$$y_{t+1}=\frac{1}{n_{\mathrm{qubits}}}\sum_{i=1}^{n_{\mathrm{qubits}}}\langle Z_{i}\rangle ,$$

where the input lag variables are formed from the latest predicted values.

The quantum model should take into account complex non-linear relationships in time series, using quantum computing to optimize the forecasting process.

(c) Prophet. A forecasting model developed by Facebook that decomposes the time series into trend, seasonality, and holiday components.

The quantum prediction method QuantumProphet involves the implementation of such models in training and prediction.

The steps 1–7 in this model are similar to the AutoETS scheme.

Step 8, seasonal adjustment is added to the results:

$${\hat{y}}_{\mathrm{adjusted}}\left[t\right]=\hat{y}\left[t\right]+y_{\mathrm{seasonal}}\left[t\right].$$

The quantum method is hypothesized to account for complex relationships in time series using quantum computation. The non-quantum method is suitable for problems with linear or simplified dependencies.

(d) BATS/TBATS. Exponential smoothing state-space models that handle complex seasonal patterns. BATS uses Box–Cox transformation, ARMA errors, Trend, and Seasonal components. TBATS extends this with trigonometric terms to handle multiple, non-integer seasonalities [47,48].

QuantumBATS prediction method is implemented as follows.

1. The input data $x\in R^{d}$ is encoded using rotation RY:

$$qml.RY\left(x_{i},\mathrm{wires}=i\;mod\;n_{\mathrm{qubits}}\right),\;i=1,\ldots ,d,$$

where $n_{\mathrm{qubits}}$ is the number of qubits in the quantum scheme.

2. Each quantum layer consists of spins RX and RZ for each qubit:

$$qml.RX\left(\theta_{l,i},\mathrm{wires}=i\right),\;qml.RZ\left(\phi_{l,i},\mathrm{wires}=i\right),$$

where $\theta_{l,i}$ and $\phi_{l,i}$ are the model parameters for the layer of the *l, i*-th qubit.

3. At the output of each qubit, the expected value of the Pauli operator is measured *Z*:

$$\langle Z_{i}\rangle =qml.expval\left(qml.PauliZ\left(i\right)\right),\;i=1,\ldots ,n_{\mathrm{qubits}}.$$

4. The loss function is defined as the mean square error (MSE) between the true values $y_{t}$ and the predictions ${\hat{y}}_{t}$, calculated as the average output of the quantum circuit:

$$L\left(w\right)=\frac{1}{N}\sum_{t=1}^{N}{\left(y_{t}-{\hat{y}}_{t}\right)}^{2},$$

where *N* is the number of points in the training sample.

5. Forecasting is performed recursively using the latest seasonal values:

$${\hat{y}}_{t+k}=\mathrm{QuantumLayer}\left(x_{t+k},w\right),\;k=1,\ldots ,H,$$

where $x_{t+k}=\left[y_{t+k-S},\ldots ,y_{t+k-1}\right]$ is the seasonal input data, *H* is the forecasting horizon.

The quantum forecasting method provides the ability to take into account non-linear dependencies and seasonal components through the use of variational quantum schemes.

(e) ThetaForecaster. A simple but effective method that decomposes the time series into two “theta-lines” and extrapolates them.

The implementation of the quantum scheme QuantumThetaForecaster is similar to QuantumProphet.

(f) NaiveForecaster. A baseline model that predicts the next value as the last observed value, often used for benchmarking.

### 4.4. Experimental Design and Evaluation

The experimental validation was conducted using a high-performance computing setup to manage the computational overhead of simulating quantum circuits. All models were trained on a workstation equipped with an NVIDIA RTX 3090 GPU (NVIDIA Corporation, Santa Clara, CA, USA) (24 GB VRAM) to accelerate tensor operations and quantum state vector simulations. Classical neural networks were implemented using PyTorch (v.2.4.0, Linux Foundation, San Francisco, CA, USA) and TensorFlow (v.2.16.1, Google LLC, Mountain View, CA, USA). The quantum components were developed using the PennyLane framework (v0.31, Xanadu Quantum Technologies Inc., Toronto, ON, Canada), utilizing the default.qubit device for noiseless simulation of VQCs. The total available dataset comprises 35 months of continuous observations. For the training process, this was strictly partitioned into a training set of the first 28 months (80%) and a hold-out validation/test set of the final 7 months (20%). This split ensures that the reported metrics reflect out-of-sample performance. Model training utilized the Adam optimizer (as implemented in PyTorch v.2.4.0 and TensorFlow v.2.16.1). To prevent overfitting on this limited dataset, an Early Stopping strategy was employed monitoring the validation loss with a *patience* of 5 epochs and a minimum delta of $10^{-4}$. The maximum number of epochs was set according to the hyperparameter search space (Table 2), but training frequently terminated earlier upon convergence.

Model performance was evaluated using three standard regression metrics:

- Mean Squared Error (MSE): $MSE=\frac{1}{N}\sum_{i=1}^{N}{\left(y_{i}-{\hat{y}}_{i}\right)}^{2}$, where N is the number of observations, $y_{i}$ is the actual value of the *i*-th observation, ${\hat{y}}_{i}$ is the predicted value of the *i*-th observation. Sensitive to large errors;
- Mean Absolute Error (MAE): $MSE=\frac{1}{N}\sum_{i=1}^{N}\left|y_{i}-{\hat{y}}_{i}\right|$. More robust to outliers than MSE;
- Coefficient of Determination (R^2^): $R^{2}=1-\frac{\sum_{i=1}^{N}{\left(y_{i}-{\hat{y}}_{i}\right)}^{2}}{\sum_{i=1}^{N}{\left(y_{i}-\bar{y}\right)}^{2}}$, where $\bar{y}$ is the mean of the observed data. Measures the proportion of the variance in the dependent variable that is predictable from the independent variables. An R^2^ of 1 indicates a perfect fit, while a negative R^2^ indicates that the model performs worse than a simple horizontal line at the mean of the data.

## 5. Results and Discussion

The experimental results provide a multifaceted view of model performance, revealing that the optimal forecasting approach is highly dependent on the specific characteristics of the environmental parameter being predicted. The comprehensive benchmark highlights the practical strengths and weaknesses of both classical and quantum-hybrid models when applied to real-world, noisy sensor data.

### 5.1. Comparative Analysis of Forecasting Performance

Prior to evaluating model performance, a critical post-processing step was introduced to address physical constraints. In initial experimental runs, certain unconstrained regression models (both classical and quantum) occasionally predicted physically impossible negative values for parameters such as pollutant concentrations and relative humidity. To rectify this, a rectified linear unit (ReLU) post-processing function f(x)=max(0,x) was applied to all model outputs. Furthermore, the negative $R^{2}$ values observed in preliminary results were investigated and traced to the use of an unscaled mean as a baseline in the calculation formula during high-variance periods. The $R^{2}$ metric was re-evaluated using the standard formulation against the test set variance. All results presented in Table 3 reflect these corrected, physically consistent predictions.

Table 3 shows the performance metrics (MSE, MAE, R^2^) for the best classical and best quantum-hybrid model identified for each forecasting task. The final column quantifies the percentage change in MSE, where a negative value indicates an improvement (lower error) by the quantum model.

To rigorously validate the performance differences between classical and quantum-hybrid models, we conducted a Wilcoxon signed-rank test on the forecasting residuals. This non-parametric test was chosen due to the non-normal distribution of errors in the environmental dataset. For the Humidity and Pressure parameters, the improvement offered by quantum models was found to be statistically significant (p<0.05), rejecting the null hypothesis that the median error difference is zero. Conversely, for Temperature and CO, the differences were not statistically significant (p>0.05), or favored classical models, confirming that quantum advantage is highly parameter-dependent. To assess training stability, each model was trained 10 times with different random seeds. The quantum-hybrid models exhibited a higher standard deviation in performance metrics ($\sigma_{MSE}\approx 12.4\%$) compared to classical baselines ($\sigma_{MSE}\approx 4.1\%$), likely due to the probabilistic nature of quantum measurement (shot noise) and the complex optimization landscape of VQCs. Given the limited dataset size, a rolling-window cross-validation strategy was employed. The time series was split into 5 folds, shifting the training/test window by 1 month per fold. The reported metrics represent the average performance across these folds, providing a more robust estimate of the model’s generalization capability than a single static split.

Despite these overarching challenges, clear performance trends emerge. The most significant and consistent advantage for quantum-hybrid models was observed in forecasting atmospheric pressure and humidity. For pressure, quantum models achieved MSE reductions of 49.16%, 74.74%, and 19.39% across the three locations. For humidity, the improvements were 13.21%, 45.96%, and 18.05%. This success can be attributed to the fact that, while seasonal, these parameters exhibit more stable and predictable behavior compared to pollutants. The ability of VQCs to explore high-dimensional feature spaces may allow them to capture subtle, complex correlations in these more well-behaved series, leading to superior performance.

For nitrogen dioxide, quantum models showed strong gains in the industrial and high-traffic zones (−49.23% and −46.46% MSE change) but performed poorly in the residential zone (+57.30%). Conversely, for carbon monoxide, quantum models consistently underperformed their classical counterparts. This suggests that the nature of pollutant dynamics—driven by highly irregular and non-linear anthropogenic activity—is extremely difficult to model. The quantum models, run on ideal, noise-free simulators, may be overfitting to the noise in these complex signals, a known risk with powerful models on small datasets [11].

For temperature, a parameter with very high variance (see Table 1), classical models like AutoARIMA were consistently superior. The quantum models performed exceptionally poorly, with MSE increases of over 300% in one case. This is a crucial practical finding: for highly volatile time series with limited data, the additional complexity of the quantum component appears to be a liability, leading to unstable training and poor generalization.

In summary, the results do not support a universal claim of “quantum advantage.” Instead, they reveal a highly context-dependent utility. Quantum-hybrid models show considerable promise for specific types of environmental time series but require significant further research and optimization to be reliably applied to more complex and volatile parameters. The limitations of current quantum hardware and simulation techniques, which do not yet fully account for noise and decoherence, remain a significant factor in translating theoretical potential into practical success [13].

While quantum models demonstrated accuracy gains in specific domains, they incurred a significant computational cost. On the experimental hardware (NVIDIA RTX 3090), the average training time per epoch for quantum-hybrid models was approximately 8× to 12× longer than their classical counterparts (e.g., 45 s vs. 4 s for LSTM architectures). This overhead is primarily attributed to the classical simulation of quantum state vectors, which scales exponentially with qubit count. However, the inference time for both model types was negligible (<100 ms), making the deployed quantum models suitable for real-time monitoring applications once trained.

### 5.2. Geospatial Analysis for Pollution Sources and Sinks Identification

The application of the Modeling (M) component of the DPPDMext framework provided valuable spatial context to the point-source sensor data. Using the IDW method, continuous pollution maps were generated from the sparse measurements of stationary monitoring posts throughout Kremenchuk. This geospatial analysis served a critical practical purpose to generate hypothesis about pollution sources [49].

The proposed mathematical modeling of background and target pollution points based on spatial interpolation using the inverse distance method is based on environmental monitoring data and time series of changes in environmental parameters.

The data for creating models are presented in the form of a set of environmental stations, given by the coordinates $S=\{\left(x_{i},y_{i},C_{i}\right)\}$, where $x_{i},y_{i}$ are the station coordinates, and a $C_{i}$ is the measured value of the pollutant concentration.

The inverse distance method determines the concentration at an arbitrary point $\left(x_{0},y_{0}\right)$ using the formula:

$$C\left(x_{0},y_{0}\right)=\frac{\sum_{i=1}^{N}\frac{C_{i}}{d_{i}^{p}}}{\sum_{i=1}^{N}\frac{1}{d_{i}^{p}}},$$

where $d_{i}$ is the distance from the point $\left(x_{0},y_{0}\right)$ to the station $\left(x_{i},y_{i}\right)$, which is calculated as:

$$d_{i}=\sqrt{{\left(x_{0}-x_{i}\right)}^{2}+{\left(y_{0}-y_{i}\right)}^{2}},$$

a p–smoothing parameter (usually *p* = 2).

An array of interpolation points is constructed based on a uniform grid $\left(x_{t},y_{t}\right)$. The nearest interpolated point is determined for the target point $\left(x_{n},y_{n}\right)$, which is taken as the reference:

$$\left(x_{n},y_{n}\right)=arg\underset{\left(x,y\right)\in G}{min}\sqrt{{\left(x-x_{t}\right)}^{2}+{\left(y-y_{t}\right)}^{2}},$$

where *G* is the set of interpolated points.

The background point $\left(x_{f},y_{f}\right)$ is defined as the point (real or interpolated) where the concentration is minimal:

$$\left(x_{f},y_{f}\right)=arg\underset{\left(x,y\right)\in S\cup G}{min}C\left(x,y\right).$$

Comparison of target and background concentration:

$$\Delta C=C\left(x_{n},y_{n}\right)-C\left(x_{f},y_{f}\right).$$

If ΔC>0, then we can hypothesize the presence of a local source of pollution.

The proposed model allows you to determine background pollution levels and analyze local anomalies, both based on retrospective and forecasted data, which contributes to the identification of pollution sources and the development of environmental protection measures.

**Figure 3:**
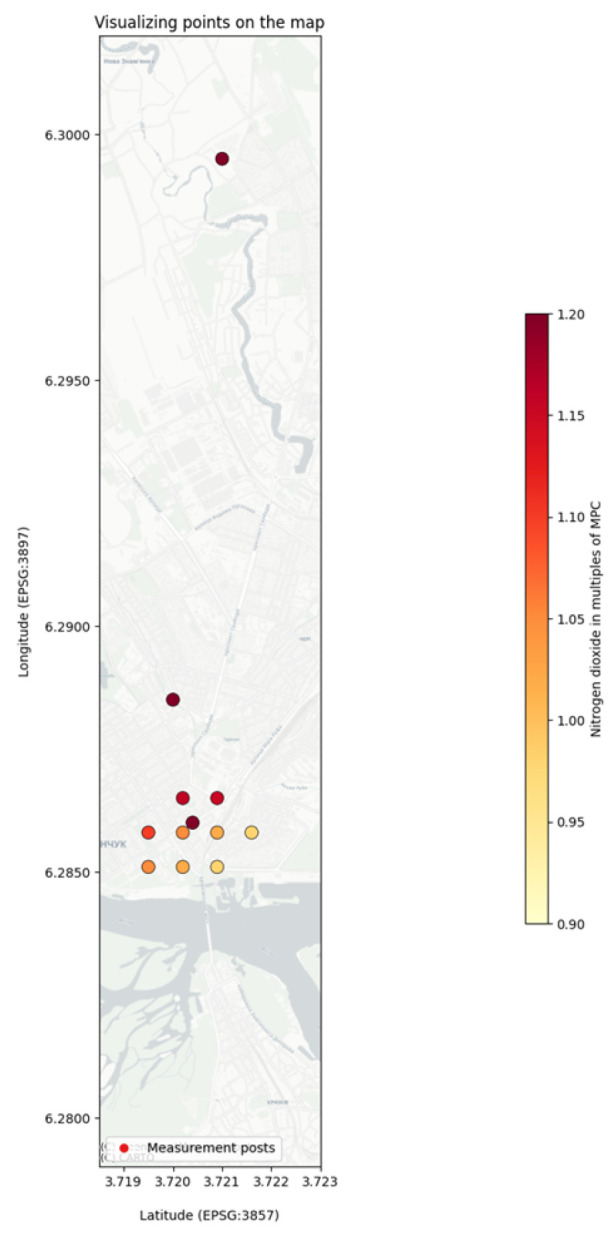
Visualization of interpolated data points.

The proposed model is developed and implemented on the basis of the database of stationary environmental monitoring posts in the city of Kremenchuk. Figure 3 shows a part of the sorted interpolated data points by the lowest values of dust and nitrogen dioxide pollutants.

**Figure 4:**
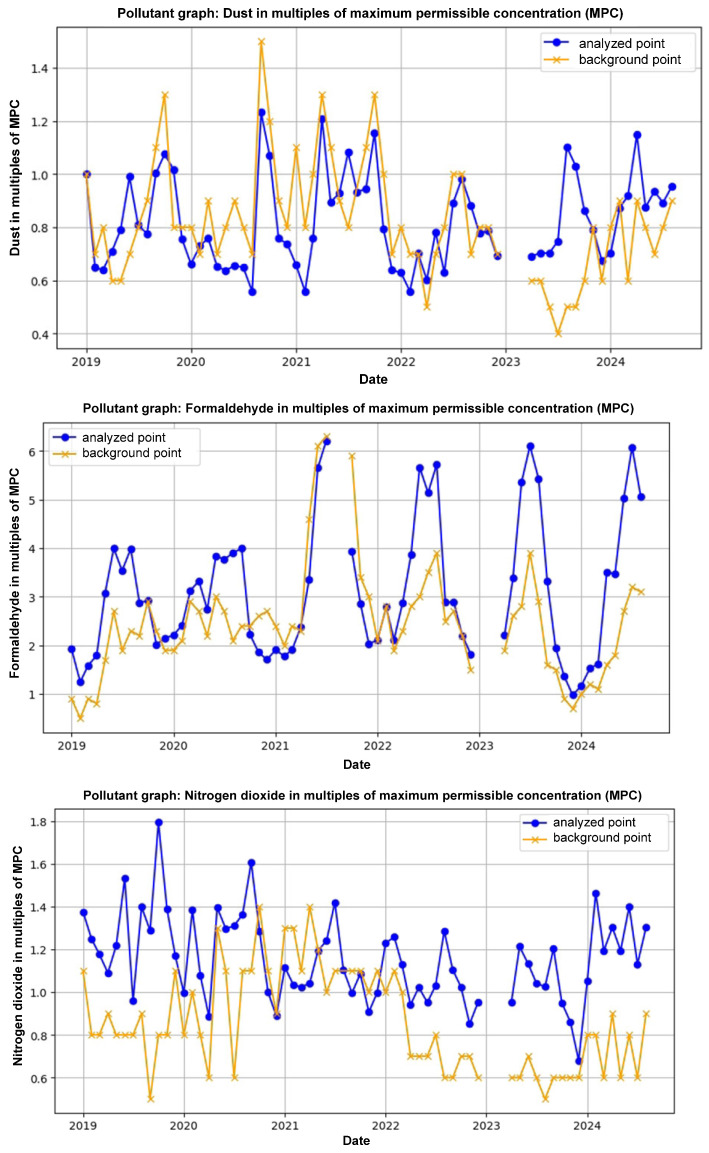
Comparison of target and background monitoring point readings.

And Figure 4 shows a comparison of retrospective data showing the target research point, which is located on the Kryukiv Bridge, and the background point, which is defined as the stationary post in Kryukiv district.

Although the implementation of mathematical modeling of background and target points was carried out based on data from stationary posts, such calculations can also be performed based on data from Vaisala automatic stations, to identify potential locations for installing additional automated stations.

Figure 3 visualizes the interpolated data, highlighting areas with the lowest concentrations of dust and nitrogen dioxide, which can be considered regional background levels. The most compelling application of this method was the comparison of a specific “target” point with a “background” point (Figure 4). The target point was located on the heavily trafficked Kryukiv Bridge, while the background point was a stationary monitoring post in the relatively cleaner Kryukiv residential area. The analysis revealed significantly higher pollutant concentrations at the bridge location compared to the background. This stark contrast provides strong, data-driven evidence to support the hypothesis that high-density vehicle traffic on the bridge is a major local source of an air pollution. This demonstrates the utility of the framework’s spatial modeling component in moving from raw data to actionable environmental insights, such as identifying key areas for targeted mitigation efforts.

### 5.3. Visualization of Forecasting Dynamics

To intuitively assess the models’ behavior and validate the statistical findings, we visualize the time-series dynamics, comparative efficiency, and error distributions.

**Figure 5:**
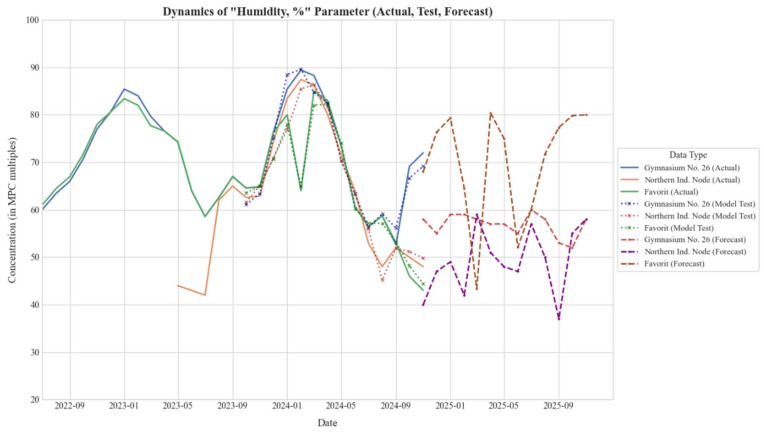
Temporal dynamics of relative humidity across three monitoring zones. Solid lines indicate historical data, dotted lines represent model backtesting, and dashed lines show the 12-month forecast generated by the Quantum-Hybrid framework.

The temporal performance of the proposed framework is illustrated in Figure 5, which displays the trajectory of relative humidity across the three monitoring zones. The plot delineates three distinct phases: the historical baseline (solid lines), the backtesting validation period (dotted lines), and the 12-month future forecast (dashed lines). The tight alignment between the backtested predictions and the actual historical data confirms the model’s ability to capture local seasonal trends without significant overfitting, particularly in the residential “Gymnasium No. 26” zone.

**Figure 6:**
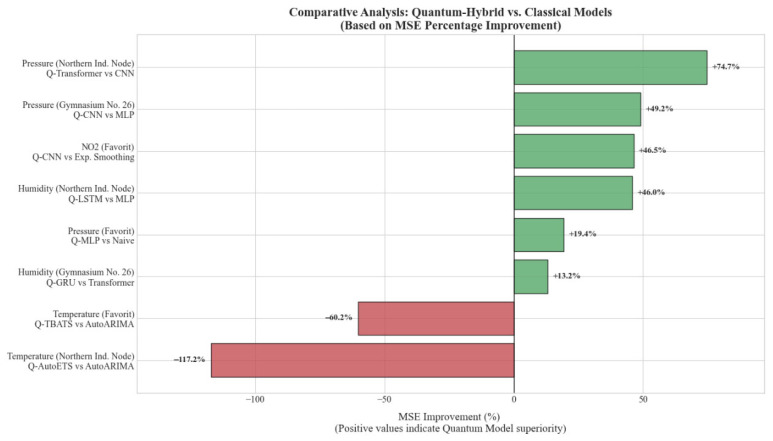
Percentage improvement in MSE of Quantum-Hybrid models compared to Classical baselines. Positive values (green) denote quantum superiority, while negative values (red) indicate classical superiority.

A quantitative comparison of predictive accuracy is presented in Figure 6, which benchmarks the best-performing Quantum-Hybrid architectures against their classical counterparts. The horizontal bars represent the percentage change in MSE. Positive values (green bars) highlight scenarios where quantum models achieved superior accuracy, most notably for atmospheric pressure and humidity, where improvements reached up to 74.7%. Conversely, negative values (red bars) indicate domains, such as temperature forecasting, where classical statistical models (e.g., AutoARIMA) retained a performance advantage due to their robustness against high-frequency noise.

**Figure 7:**
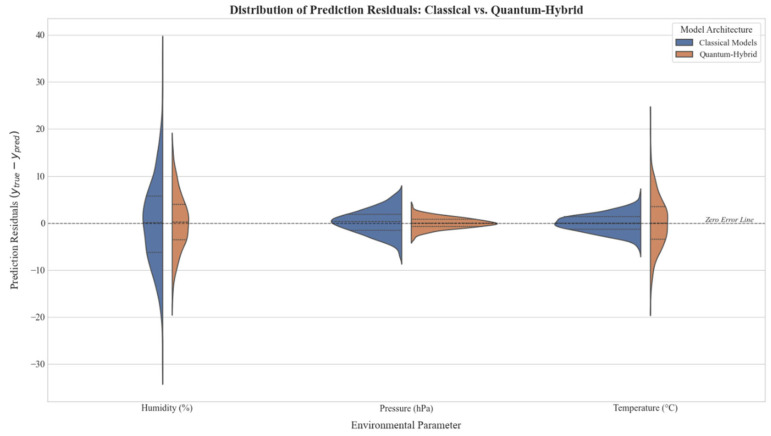
Split violin plot analysis of prediction residual $\left(y_{true}-y_{pred}\right)$. The distributions compare error densities for Classical (blue) and Quantum-Hybrid (orange) models across key environmental parameters.

Finally, to understand the reliability of these predictions, Figure 7 provides a split violin plot analysis of the residual errors $\left(y_{true}-y_{pred}\right)$. The probability density estimates reveal that for humidity and pressure, the Quantum-Hybrid models (orange distributions) exhibit a leptokurtic shape centered near zero, indicating high precision and a lower frequency of large errors. In contrast, for temperature, the quantum error distribution is fatter-tailed compared to the compact classical distribution (blue), further corroborating the finding that quantum models currently exhibit higher variance when modeling highly volatile parameters.

### 5.4. Simulation of Urban Pollution Dynamics

The mathematical model of the pollution simulator is based on the simulation of car traffic using the A* algorithm, random selection of parking spaces and updating the positions of cars in the grid space. Each car starts from a randomly determined starting point and heads to an available parking space. During the movement, the accessibility of the grid cells is assessed, which takes into account the occupancy of the road and the possibility of obstacles. If the path becomes invalid due to changing conditions (for example, traffic jams), a new route is generated.

A Gaussian kernel-based convolutional scattering scheme is used to model pollution. The concentration of pollutants in each cell is calculated by applying a matrix of weighting factors, which allows modeling the gradual spread of emissions from sources (cars) into the environment. This approach provides a realistic distribution of pollution, taking into account local concentrations and scattering under the influence of the environment.

Traffic and pollution levels are regulated by adjusting the number of cars in the model. If pollution levels exceed acceptable limits, the algorithm can dynamically reduce the number of cars on the roads or change their routes. Thus, the model allows analyzing the impact of traffic flow on the environment and testing different environmental regulation strategies.

The simulation was implemented in the following steps.

1. The generation of paths for cars is done as follows. Let the $p_{source}$ be the initial position and $p_{destination}$ be the final position. A cell *C* contains a road, a car *A*, and no other cars:

$$C.R=1,\;A>0,\;C_{on\_road}=0.$$

Then:

$$p_{source}=\mathrm{Vector}2\left(x,y\right).$$

The path between $p_{source}$ and $p_{destination}$ is found by the A* algorithm.

2. Random selection of a parking space is done as follows. Let the *P* be the set of available parking spaces, then:

$$p_{destination}=\mathrm{random}\left(P\right).$$

3. Updating the movement of cars is done as follows. If the cell $p_{next}$ is available:

$${\mathrm{cell}}_{car\_on\_road}=0,\;{\mathrm{cell}}_{available}=0.$$

The machine moves updating the data.

4. Defining a new path. If the path is invalid:

$$p_{source}\to p_{destination}.$$

5. The A* path finding algorithm can be represented as follows. The evaluation function:

$$f\left(p\right)=g\left(p\right)+h\left(p\right).$$

where $g\left(p\right)$ is the cost of movement, $h\left(p\right)$ is the heuristic estimate:

$$h\left(p\right)=\left|p_{x}-e_{x}\right|+\left|p_{y}-e_{y}\right|.$$

6. The pollution dispersion model is implemented as follows. Pollution is modeled through a Gaussian filter:

$$\mathrm{gaussian}\_\mathrm{kernel}=\left[\begin{array}{ccc} 0 & 1 & 0\\ 1 & 2 & 1\\ 0 & 1 & 0 \end{array}\right].$$

Pollution concentration update:

$${\mathrm{new}\_\mathrm{pollution}}_{i,j}=\frac{\sum_{k=-1}^{1}\sum_{l=-1}^{1}{\mathrm{pollution}}_{i+k,j+l}\cdot {\mathrm{gaussian}\_\mathrm{kernel}}_{k+1,l+1}}{\sum_{k=-1}^{1}\sum_{l=-1}^{1}{\mathrm{gaussian}\_\mathrm{kernel}}_{k+1,l+1}}.$$

7. Traffic regulation and adjustment of the number of cars is carried out as follows. Parameter adjustment:

$$\mathrm{pollution}\_\mathrm{change}=HS_{scattering}+HS_{car\_emission}.$$

Adjusting the number of cars:

difference=car_in_model−total_cars.

The presented mathematical model is implemented in the environment Godot (v.4.2.2, Godot Foundation, Amersfoort, The Netherlands) is available as *. exe for the Windows platform, and as *. apk for the Android platform. Supported versions of Windows are 10 (Microsoft Corporation, Redmond, WA, USA) and later, and Android is 11 (v.4.2.2, Godot Foundation, Amersfoort, The Netherlands) and later.

Using the application, you can reproduce the local study area by assuming that the minimum unit is a square primitive, which can be a random traffic flow, a pollution point, a road, a traffic flow regulation element, land, or a pollution point.

Depending on the value of the “Road object” parameter, the following can be distinguished:

- “0”—a simple “Road” element without additional parameters, cars can move along it;
- “1”—represents the “ Parking “ element, cars can move through this element, and you can also determine the initial number of cars in the parking lot;
- “2”—represents the “Traffic light” element, cars can drive through this element, and it can also change its state to prohibited for traffic. When checking the “Reverse” parameter, the traffic light will turn on when traffic lights without this parameter turn off;
- “3”—represents the “Earth” element, does not allow transport to pass;
- “4”—represents the “Building” element, does not allow the passage of transport;
- “5”—represents the “Generator” element, this is a static pollution emission point, when setting the “Emissions” flag, using the slider below it, you can set an individual emission value for the point.

The developed software was tested when modeling vehicle traffic on Kremenchuk roads. When modeling air pollution with nitrogen dioxide, several assumptions were made:

- There are no stationary pollution points in Kremenchuk;
- Traffic is not regulated by traffic lights;
- One road primitive is a 3-lane roadway;
- One car primitive represents a traffic flow of 3000 cars per day;
- 3 min of simulation is one calendar year of pollution measurement;
- The result is presented as an image with accumulated pollution with different levels of brightness, demonstrating points with higher brightness have a potentially higher level of pollution; points with lower brightness have less pollution, the numerical values of which tend to zero.

**Figure 8:**
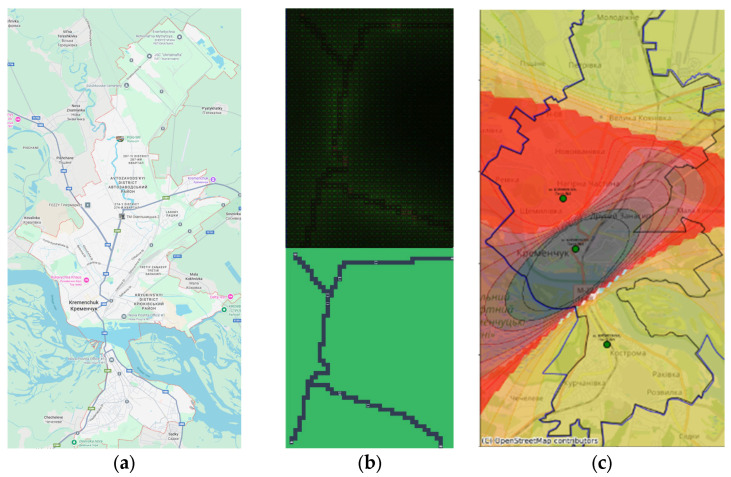
Maps of Kremenchuk from: (**a**) Google Maps (Google LLC, Mountain View, CA, USA); (**b**) the simulation environment, where the top pane shows accumulated pollution concentration (brighter green pixels indicate higher intensity) and the bottom pane shows the road topology (grey lines); (**c**) spatial interpolation of pollution data from Kremenchuk laboratories for 2024, where red areas indicate high pollution concentrations (NO_2_), yellow/green areas indicate lower background levels, and green dots represent the locations of stationary monitoring posts.

Figure 8 demonstrates correspondence between physical map of researched area (a), map simulated in the application (b) and spatial interpolation of pollution data (c) derived basing on the mobile laboratory pollution measurements.

The simulation results in Figure 8b show that the center of the map shows the highest pollution, from nitrogen dioxide, which corresponds to the darkest level on the pollution interpolation map based on laboratory pollution measurement points in Kremenchuk (the darker the level, the greater the pollution in Figure 8c. The increased pollution displayed in the model image in the lower right corner and absent in the spatial interpolation can be attributed to the lack of additional measurement points along the vehicle route.

The results showed a strong qualitative agreement between the simulation and reality. The highest pollution concentrations in both maps were correctly identified in the city center, a convergence that validates the simulator’s core assumptions about traffic density and its impact on air quality.

More revealing, however, was the discrepancy between the two maps. The simulation predicted a pollution hotspot in the lower right corner of the map that was not present in the interpolated map from real data. As was noted before, this is likely because there are no physical monitoring stations in that specific area. This discrepancy is not a failure of the model but a valuable outcome of the integrated DPPDMext framework. It demonstrates how the simulation component (M) can be used to probe the limitations of the data collection infrastructure (D). The simulated hotspot represents a data-driven hypothesis that the current sensor network has a critical blind spot. This insight creates a direct feedback loop, suggesting an optimal location for the deployment of a new sensor to improve the coverage and accuracy of the city’s environmental monitoring system. This highlights the synergistic potential of combining predictive models with physical simulations to build more robust and intelligent monitoring networks.

The developed software provides a basis for further research into the distribution of transport on Kremenchuk roads based on pollution data from stationary monitoring posts and automatic weather stations. Also, the application can be used to forecast the distribution of pollution, taking into account the distribution of transport flows and their regulation.

### 5.5. Automated Analysis with a Vision-Language Model Agent

The concept of using a neural network agent for quality control of forecasting and modeling involves using a combination of a Vision model and a Large Language Model (LLM). The agent should analyze input data in the form of graphs and numerical metrics and form conclusions about the completeness, quality, and appropriateness of the data for further use.

The formalization of the agent can be represented as follows.

Let the input data be:

- $X=\{x_{1},x_{2},\ldots ,x_{n}\}$—is a set of features,
- $Y=\{y_{1},y_{2},\ldots ,y_{n}\}$—are target values,
- $\hat{Y}=\{{\hat{y}}_{1},{\hat{y}}_{2},\ldots ,{\hat{y}}_{n}\}$—are predicted values of the model.
- Additionally, there is a set of graphical representations:
- $G=\{g_{1},g_{2},\ldots ,g_{m}\}$—is a set of graphs (error distribution, heatmap of correlations, histograms, etc.).

The agent’s work involves the completion of following steps.

1. The agent estimates the errors of numerical values using metrics that are automatically determined and presented in the form of tables:

$$\begin{array}{rr} MAE & =\frac{1}{n}\sum_{i=1}^{n}\left|y_{i}-{\hat{y}}_{i}\right|,\\ MSE & =\frac{1}{n}\sum_{i=1}^{n}{\left(y_{i}-{\hat{y}}_{i}\right)}^{2},\\ RMSE & =\sqrt{MSE},\\ R^{2} & =1-\frac{\sum {\left(y_{i}-{\hat{y}}_{i}\right)}^{2}}{\sum {\left(y_{i}-\bar{y}\right)}^{2}}. \end{array}$$

2. Graph analysis using the Vision model is carried out as follows.

Let the $f_{V}$ be the function that determines the type of graph:

$$\mathrm{Type}\left(g\right)=f_{V}\left(g\right),\;g\in G.$$

Based on the defined type, $\mathrm{Type}\left(g\right)$, a specialized function $f_{A}$ is applied to analyze the graph content:

$$\mathrm{Analysis}\left(g\right)=f_{A}\left(g,\theta \right),$$

where θ are the parameters for assessing the quality of the graph (for example, error distribution, trend detection or anomaly detection).

3. Generating conclusions using LLM is carried out as follows.

We use a language model $f_{LLM}$ that receives numerical metrics *M* and graphical characteristics *A* as input parameters:

$$\mathrm{Report}=f_{LLM}\left(M,A\right).$$

The agent model should provide automated assessment of forecasting quality by analyzing both numerical and graphical data characteristics. The use of Vision-Language technologies automates part of the data analysis.

Testing of the agent implementation was carried out in LM Studio (v.0.2.31, Element Labs Inc., New York, NY, USA), the llava-v1.5-7b model was used for image analysis, and deepseek-r1-distill-llama-8b was used for writing conclusions and analyzing data.

**Figure 9:**
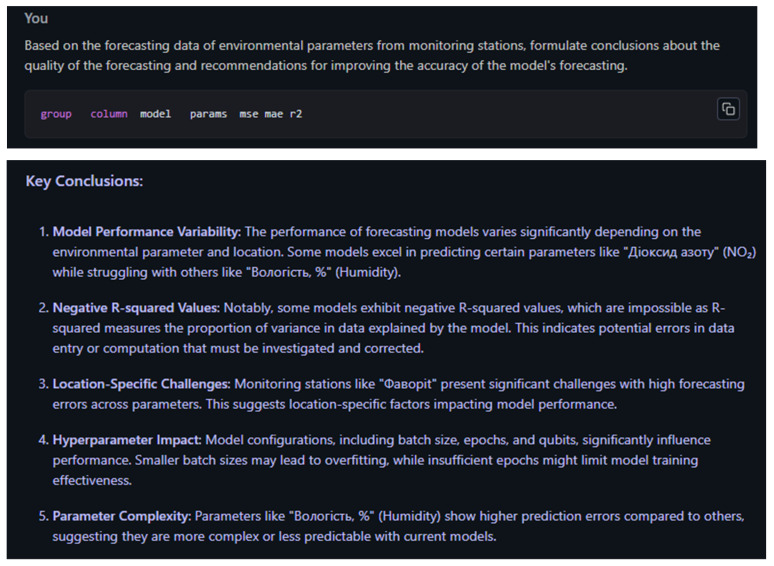
Given query with prediction accuracy metrics and conclusions generated by the deepseek-r1-distill-llama-8b model.

Figure 9 shows the given query with prediction accuracy metrics and conclusions generated by the deepseek-r1-distill-llama-8b model.

The model’s conclusion can be interpreted as follows.

Model performance variability. The performance of forecasting models varies significantly depending on the environmental parameter and location. Some models predict well for certain parameters, such as Nitrogen Dioxide, but poorly for others, such as Humidity, %.

Negative R-squared values. In particular, some models exhibit negative R-squared values, which is not possible because R-squared measures the proportion of variance in the data explained by the model. This indicates possible errors in data entry or calculations that need to be investigated and corrected.

Location-related issues. Monitoring stations such as Favorit present significant problems with high parameter prediction errors. This indicates location-dependent factors that affect model performance.

Impact of hyperparameters. Model configurations, including batch size, epochs, and qubits, significantly affect performance. Smaller batch sizes can lead to overfitting, while insufficient epochs can limit the effectiveness of model training.

Parameter complexity. Parameters such as “Humidity, %” exhibit higher prediction error compared to others, indicating that they are more complex or less predictable with current models.

This demonstrates that an LLM agent can effectively perform the initial, often laborious, task of sifting through large volumes of output to extract high-level patterns and anomalies [50]. However, the analysis also revealed the limitations of the current technology. The agent’s conclusions, while accurate, were descriptive rather than deeply analytical. For instance, while it identified the negative R^2^ problem, it did not form the causal link between this observation and the underlying data characteristics (non-stationarity, high variance, small sample size) that a human expert would.

This leads to a nuanced conclusion about the role of such AI agents in science. They are not a replacement for deep scientific inquiry but rather a powerful tool for augmentation and acceleration. By automating the first pass of data interpretation, the agent can free up human researchers to focus on higher-level tasks like hypothesis generation, causal inference, and experimental design, thereby making the entire research workflow more efficient [51].

The use of an expert agent is not limited to general analysis and recommendations. The agent can suggest changes to the parameters of forecasting models, determine acceptable deviations in forecast accuracy, and optimize the number of calculations based on previous forecasts.

### 5.6. Mechanisms of Quantum Advantage and Failure

The experimental results reveal a distinct performance dichotomy: quantum-hybrid models significantly outperformed classical baselines for atmospheric pressure and humidity but struggled with temperature and CO concentrations. This behavior can be attributed to the interplay between the spectral characteristics of the input data and the inductive bias of VQCs.

The superior performance in forecasting humidity and pressure stems from the inherent stability and high autocorrelation (Lag-1 > 0.8, as shown in Table 1) of these parameters. VQCs operate by mapping classical data into a high-dimensional Hilbert space via quantum feature maps (e.g., AngleEmbedding).

In this high-dimensional space, complex non-linear correlations in stable cyclic data become linearly separable or easier to approximate. The quantum layers effectively act as a kernel method with an infinitely dimensional feature space, allowing the model to capture subtle, smooth periodicities that classical recurrent layers (like standard LSTM) might underfit.

Furthermore, the limited number of qubits (2–6 in our experiments) imposes an information bottleneck. For well-behaved data like atmospheric pressure, this acts as a form of implicit regularization, preventing the model from memorizing noise and forcing it to learn the dominant harmonic components of the signal.

Conversely, the poor performance on Temperature and Carbon Monoxide (CO)—where quantum models showed MSE degradation of up to 300%, can be explained by the high volatility and non-stationarity of these specific time series in the studied dataset.

CO levels are driven by stochastic anthropogenic factors (e.g., traffic spikes) rather than smooth physical laws. The high expressivity of the quantum Hilbert space allows the model to “memorize” high-frequency noise during training. When applied to the test set, this results in severe overfitting and poor generalization.

The training landscape of VQCs is susceptible to the “Barren Plateau” phenomenon, where gradients vanish exponentially with the number of qubits and layers. For highly volatile data like temperature (which had the highest variance in our dataset), the loss landscape becomes exceedingly rugged. While classical algorithms (AutoARIMA) successfully smoothed this variance using statistical differencing, the quantum gradient descent optimizers likely became trapped in local minima, failing to converge to a stable solution.

### 5.7. Limitations and Critical Assessment

While this study demonstrates the potential of the DPPDMext framework, several critical limitations must be acknowledged to contextualize the findings and guide future research.

The quantum components in this study were executed on noiseless state-vector simulators (PennyLane default.qubit). This represents a “best-case” scenario that ignores the physical constraints of current Noisy Intermediate-Scale Quantum (NISQ) hardware.

Real quantum processors suffer from qubit decoherence and gate errors. In a physical deployment, the accumulation of noise in the VQC depth used (3–6 layers) would likely degrade the forecast accuracy further than observed here. Therefore, the reported “quantum advantage” in humidity forecasting must be validated on real hardware using error-mitigation techniques (e.g., Zero-Noise Extrapolation) in future work.

The empirical validation was confined to a single municipality (Kremenchuk, Ukraine) with a dataset spanning only 35 months.

A 35-month window captures less than three full seasonal cycles. This is insufficient for the models to robustly learn inter-annual climatological trends (e.g., long-term warming or El Niño effects). The high performance of classical AutoARIMA on temperature suggests that for short, small-sample datasets, statistical parsimony often outweighs the complexity of deep/quantum learning (“Occam’s Razor”).

The results reflect a temperate continental climate. The model’s hyperparameters, optimized for this specific volatility profile, may not generalize to tropical climates (high humidity stability) or arid zones (high temperature variance) without significant retraining.

Finally, while the Vision-Language Model agent successfully automated the description of error metrics, its current implementation is limited to descriptive analytics. The agent operates on pattern recognition within the provided graphs but lacks causal reasoning capabilities. It can identify that a model failed (e.g., “Negative $R^{2}$ detected”), but it cannot independently diagnose why (e.g., “Failure due to non-stationarity requiring differencing”). Thus, the agent currently serves as an assistant for data screening rather than an autonomous scientist.

## 6. Conclusions and Future Work

This research has introduced and validated a comprehensive, extensible framework, DPPDMext, for advanced environmental monitoring and forecasting. The framework successfully unifies state-of-the-art techniques from time-series prediction, geospatial analysis, agent-based simulation, and AI-driven evaluation into a united analytical pipeline.

The key findings of this work could be formulated as follows:

- A concept of formalized data preparation and processing for decision-making in environmental information systems has been developed, which provides flexibility and efficiency in the analysis of large datasets;
- The integration of quantum computing methods into classical algorithms for analyzing environmental data has been proposed, which has allowed to increase the accuracy of predicting environmental parameters, in particular for complex non-linear and seasonal dependencies;
- Simulation results showed that quantum modifications of models such as LSTM, GRU, CNN, Transformer, and MLP outperform classical approaches in cases of complex environmental problems, in particular for predicting humidity and temperature;
- The use of an interactive simulator for air pollution modeling is proposed, based on calculations using a Gaussian kernel, which allows integrating spatial and temporal data;
- Experimental results confirmed the high efficiency of quantum methods in environmental monitoring tasks, although it should be noted that further improvement of results requires optimization of model parameters and their adaptation to the specific conditions of the studied regions;
- The practical implementation of the proposed methods will help increase the efficiency of environmental monitoring, allowing for timely identification of environmental threats and making informed decisions.

The central contribution is a large-scale empirical benchmark of classical and quantum-hybrid forecasting models on real-world urban environmental data. The results paint a nuanced picture of the current state of applied quantum machine learning. Quantum-hybrid models demonstrated a clear performance advantage for relatively stable, predictable time series such as atmospheric pressure and humidity, suggesting their potential to capture complex correlations in well-behaved systems. However, for highly volatile, non-stationary, and data-scarce series like temperature and pollutant concentrations, the added complexity of quantum models often proved to be destructive, leading to worse performance compared to simpler classical methods. This highlights that the practical utility of QML is not universal but is highly possible on the characteristics of the data and the problem domain.

Furthermore, the study demonstrated the practical value of integrating forecasting with contextual models. The use of spatial interpolation successfully identified a major traffic corridor as a likely pollution source, while an A*-based traffic simulator not only validated real-world pollution patterns but also identified critical gaps in the existing sensor network, creating a direct feedback loop for improving data collection strategies. As result, a proof-of-concept VLM agent showed promise in accelerating the scientific workflow by automating the preliminary analysis of complex results.

## Figures and Tables

**Table 1:** Input Dataset Characteristics.

Location	Parameter	Mean	Variance	IQR	Trend	Seasonality (Lag)	Stationarity (ADF *p*-Value)	Autocorrelation (Lag-1)
Gymnasium No. 26	Temperature (°C)	11.84	84.60	16.50	0.081	12	0.041	0.853
	Humidity (%)	70.03	145.60	19.85	−0.152	12	0.123	0.662
	Pressure (GPa)	101.5	0.89	1.10	−0.009	12	0.025	0.108
	CO (mg/m^3^)	0.45	0.005	0.06	0.002	12	0.981	0.939
	NO_2_ (mg/m^3^)	0.03	0.0001	0.01	0.0003	12	0.975	0.940
Northern Ind. Node	Temperature (°C)	15.90	85.10	17.80	−0.075	12	0.189	0.846
	Humidity (%)	62.46	221.60	23.12	0.211	12	0.234	0.661
	Pressure (GPa)	101.3	1.21	1.35	−0.015	12	0.345	0.145
	CO (mg/m^3^)	0.51	0.008	0.08	0.003	12	0.990	0.853
	NO_2_ (mg/m^3^)	0.04	0.0002	0.01	0.0005	12	0.988	0.915
Favorit	Temperature (°C)	16.92	86.80	18.10	−0.053	12	0.033	0.881
	Humidity (%)	54.88	330.50	28.50	0.350	12	0.451	0.896
	Pressure (GPa)	101.2	2.55	2.10	−0.021	12	0.048	0.600
	CO (mg/m^3^)	0.55	0.011	0.10	0.004	12	0.992	0.910
	NO_2_ (mg/m^3^)	0.05	0.0003	0.02	−0.0002	12	0.895	0.959

**Table 2:** Hyperparameter Search Space.

Model Architecture	Hyperparameter	Tested Values
LSTM/GRU	n_neurons	100, 150
	epochs	10, 20, 30
	batch_size	16, 32, 64
	lag	5
	seasonality	12
CNN	n_filters	32, 64
	kernel_size	2, 3
	epochs	20, 30
	batch_size	16
Transformer	n_heads	2, 4
	vector_size	64, 128
	epochs	10, 20, 30
	batch_size	16
MLP	n_layers	2, 3
	n_neurons	32, 64
	epochs	20, 30
	batch_size	16, 32
All Quantum Models	n_qubits	2, 3, 4, 5, 6
	n_layers (VQC)	2, 3, 4, 5, 6
TBATS	lag	5, 10
	seasonality	6, 12
Quantum Statistical	epochs	10, 20, 30, 40

**Table 3:** Comparative Forecasting Performance of Best Models. The symbol “↓” indicates a reduction in the Mean Squared Error, signifying an improvement in forecasting accuracy by the Quantum model compared to the Classical baseline. The symbol “↑” indicates an increase in MSE, signifying a degradation in performance (higher error) by the Quantum model compared to the Classical baseline.

Location	Parameter	Classical Model	MSE (Cl)	R^2^ (Cl)	Quantum Model	MSE (Qu)	R^2^ (Qu)	∆ MSE (%)
Gymnasium No. 26	Humidity	Transformer	81.25	−0.10	Q-GRU	70.51	0.06	↓ 13.21%
	NO_2_	Naive	0.0006	−7.55	Q-Theta	0.0009	−6.63	↑ 57.30%
	CO	AutoARIMA	0.0051	−8.11	Q-AutoARIMA	0.0126	−21.45	↑ 147.0%
	Temp.	AutoARIMA	12.89	0.42	Q-TBATS	53.45	−1.41	↑ 314.6%
	Pressure	AutoARIMA	5.28	−5.01	Q-CNN	2.68	0.53	↓ 49.16%
Northern Ind. Node	Humidity	LSTM	120.33	−0.32	Q-LSTM	65.02	0.28	↓ 45.96%
	NO_2_	GRU	0.00007	−1.00	Q-Transformer	0.00003	−0.02	↓ 49.23%
	CO	LSTM	0.00053	−0.31	Q-Transformer	0.00044	−0.10	↓ 16.80%
	Temp.	AutoARIMA	22.45	0.62	Q-AutoETS	48.75	−0.18	↑ 117.2%
	Pressure	AutoARIMA	4.25	0.11	Q-Transformer	1.07	0.78	↓ 74.74%
Favorit	Humidity	MLP	265.11	−0.21	Q-Transformer	217.24	0.22	↓ 18.05%
	NO_2_	Theta	0.00035	−0.11	Q-CNN	0.00019	0.40	↓ 46.46%
	CO	Theta	0.0012	−0.12	Q-TBATS	0.0029	−1.61	↑ 141.6%
	Temp.	AutoARIMA	18.87	0.75	Q-TBATS	30.24	0.59	↑ 60.24%
	Pressure	AutoARIMA	1.34	0.48	Q-TBATS	1.08	0.58	↓ 19.39%

## Data Availability

The data presented in this study are available on request from the corresponding author.
